# Assessing Implicit Sex and Gender Bias in Frontotemporal Dementia

**DOI:** 10.1002/alz70860_105363

**Published:** 2025-12-23

**Authors:** Jainjayne Daniels, Medha Reddy, Kaitlin Seibert, Ollie Fegter, Elena Badillo‐Goicoechea

**Affiliations:** ^1^ University of Chicago, Chicago, IL, USA; ^2^ Northwestern University Feinberg School of Medicine, Chicago, IL, USA

## Abstract

**Background:**

Frontotemporal dementia (FTD) is a neurodegenerative disease that occurs around age 40‐60 and affects behavior more than cognition. In this systematic review and meta‐analysis, we investigate the reported prevalence of FTD in female as compared to male patients and the influence of diagnostic criteria and geographic location.

**Method:**

In this systematic review and meta‐analysis, according to PRISMA guidelines, we searched the Cochrane Library, PubMed, and Embase databases for articles including keywords “frontotemporal dementia” or “behavioral variant frontotemporal dementia” AND “gender” or “sex.” Studies were included if they reported the prevalence of FTD and were available in English. Review articles and case reports were excluded. A random effects model of multivariate meta‐regression was used to identify whether female FTD prevalence varied as a function of diagnostic criteria or country.

**Result:**

A total of 31 studies were included in our meta‐analysis. We found that, overall, there were no statistically significant differences in the documented prevalence of FTD between males and females. However, a higher female prevalence of FTD was noted when diagnosing with Lund‐Manchester criteria as compared to a clinical diagnosis by clinical criteria for FTD were used (0.159, 95%CI 0.01‐0.307, *p* <0.05). Some differences in female FTD prevalence were found based on study country, with a statistically significant lower female prevalence of FTD in the Netherlands (‐0.167, 95%CI ‐0.29–0.045, *p* <0.05). Italy (‐0.021, 95%CI ‐0.123–0.081, *p* = 0.691), Sweden (‐0.058, 95%CI ‐0.264–0.148, *p* = 0.581), and the United States (‐0.095, 95%CI ‐0.236‐0.046, *p* = 0.186) were associated with lower female prevalence of FTD than other countries, although the differences were not statistically significant.

**Conclusion:**

According to our systematic review and meta‐analysis, there was no significant difference in gender prevalence in FTD. Larger cohort data of pathologically or genetically confirmed FTD is needed to further explore the gender prevalence of the FTD and the influence of geographic and cultural factors on diagnosis according to currently available diagnostic criteria.

